# Multi-omics integration of gut–skin axis in probiotic-treated dermatological conditions

**DOI:** 10.3389/fcimb.2026.1834120

**Published:** 2026-07-13

**Authors:** Yuqin Zhang, Qingye Zhang, Min Wang, Zhi Pan, Ke Xu, Jingsheng Tian, Yongmei Li

**Affiliations:** 1 Department of Dermatology and Ulcer, Gansu Provincial Hospital of Traditional Chinese Medicine, Lanzhou, Gansu, China; 2 Clinical College of Traditional Chinese Medicine, Gansu University of Traditional Chinese Medicine, Lanzhou, Gansu, China

**Keywords:** gut–skin axis, inflammatory cytokines, multi-omics integration, probiotic metabolites, short-chain fatty acids, skin barrier function

## Abstract

**Introduction:**

The gut–skin axis is an integrated biological communication network linking gut microbial metabolites, immune regulation, oxidative stress responses and skin barrier function. Dysregulation of inflammatory and metabolic pathways has been associated with dermatological disorders including psoriasis, eczema and atopic dermatitis. Postbiotic metabolites derived from probiotics are increasingly recognized for their anti-inflammatory, antioxidant and immunomodulatory activities that may contribute to skin homeostasis. Here, we aimed to investigate the multi-omics effects of probiotic metabolite extracts (PMEs) in an *in vitro* inflammatory keratinocyte model relevant to gut–skin axis-associated signaling pathways.

**Methods:**

HaCaT keratinocytes and Human Dermal Fibroblast (HDF) cells were employed as complementary *in vitro* skin models and stimulated with lipopolysaccharide (LPS) to induce an inflammatory microenvironment. The inclusion of HDF cells enabled assessment of probiotic metabolite effects on both epidermal and dermal cellular responses relevant to skin homeostasis and repair. For each experiment, triplicate biological replicates (n = 3) were used. The multi-omics profiling included LC–MS/MS-based metabolomics, RNA-seq transcriptomics, qRT-PCR validation, ELISA-based cytokine quantification, oxidative stress assays and integrated correlation network analysis using weighted gene co-expression network analysis (WGCNA).

**Results:**

After PME treatment, keratinocyte viability was significantly higher (95.4%) compared with the inflammatory control group (62.4%). Pro-inflammatory cytokine levels were significantly decreased (TNF-α: 86.7 to 28.9 pg/mL; IL-6: 79.5 to 25.7 pg/mL), intracellular ROS decreased from 248 RFU to 116 RFU, and antioxidant enzyme activities were restored. Metabolomic profiling showed increased levels of short-chain fatty acids (SCFAs) such as butyrate (1.24 to 3.02 µmol/g), which are beneficial to the skin. Transcriptomic and qRT-PCR analyses showed upregulation of genes associated with the skin barrier, such as FLG (1.28-fold), LOR (1.31-fold) and IVL (1.29-fold).

**Discussion:**

Integrated multi-omics analysis suggested that PMEs may modulate inflammatory signaling and enhance epidermal barrier-related gene expression through coordinated metabolic and transcriptional regulation, which may validate the therapeutic potential of probiotic-derived metabolites as candidate adjunctive strategies for inflammatory skin disorders.

## 1 Introduction

The human skin, the body’s largest organ, serves as a critical barrier against environmental stressors, pathogens, and harmful chemicals while maintaining immune and physiological homeostasis (Jiao et al., 2024). Disruption of skin barrier integrity and immune regulation can lead to various dermatological conditions, including psoriasis, atopic dermatitis, eczema, and acne (Baker et al., 2023). The gut-skin axis, a complex bidirectional communication pathway that links the gut microbiota, immune system, metabolic pathways, and skin health, has been gaining attention in recent years (Hu et al., 2024). Disruption of gut microflora and microbial metabolites has been verified to affect systemic inflammation and immunity, which may promote the onset and progression of inflammatory skin diseases (Arifuzzaman et al., 2024).

Microbial metabolites, such as short-chain fatty acids (SCFAs), indole derivatives, and bile acid metabolites, produced by the gut microbiota are critical for regulating host metabolism, immune function, and epithelial barrier integrity (Yao et al., 2024). These metabolites are signaling molecules that modulate immune cell activity, inflammation, and epithelial differentiation (Baker and Rutter, 2023). Dysbiosis of the gut microbiome, defined as a decline in the diversity of resident flora and an increase in pathobionts, has been linked to various inflammatory diseases, including skin diseases (Zhang et al., 2024). Thus, restoring microbial equilibrium with dietary amendments, probiotics, or microbial metabolites has become a viable therapeutic approach to positively alter skin health (De Almeida et al., 2023).

Probiotics are defined as live microorganisms that, when administered in adequate amounts, confer health benefits on the host (Parichat and Pongsak, 2023). Beyond the effects attributable to live bacterial cells, recent investigations have highlighted the role of postbiotics - bioactive metabolites produced during microbial fermentation - as key mediators of probiotic health benefits (Hijová, 2024). Such metabolites have anti-inflammatory, antioxidant, and immunomodulatory properties and can affect host physiological processes in the absence of viable bacterial cells. Probiotic metabolites, including butyrate and indole derivatives, have been shown to attenuate activated inflammatory signaling pathways, improve epithelial barrier function, and regulate host gene expression associated with immune modulation and tissue repair (Meng et al., 2024).

Despite growing interest in the gut–skin axis, the molecular mechanisms through which probiotic-derived metabolites regulate host inflammatory responses and skin barrier function remain incompletely understood (Jimenez-Sanchez et al., 2025). The recent development of next-generation sequencing (NGS) technologies, combined with other high-throughput omics approaches such as metabolomics and transcriptomics, enables a comprehensive investigation of the interplay between the microbiome, its bioactive products, and the host molecular pathways they influence (Cuciniello et al., 2023). Most previous studies have primarily focused on microbiome composition, individual metabolites, or isolated molecular pathways, often using single-omics approaches that provide only a partial understanding of host–microbiome interactions. Furthermore, many investigations have relied on clinical associations or *in vivo* observations, limiting direct mechanistic evaluation of metabolite-mediated cellular responses (Nwosu and Popova, 2025). An integrated multi-omics strategy combining metabolomics and transcriptomics within a controlled inflammatory keratinocyte model offers a unique opportunity to systematically investigate how probiotic metabolites influence inflammatory signaling, oxidative stress responses, and epidermal barrier-associated gene expression. By linking metabolite alterations with transcriptional regulation, this approach enables a systems-level characterization of gut–skin axis communication and provides mechanistic insights that extend beyond conventional single-omics studies.

Therefore, the present study aimed to investigate the multi-omics integration of the gut–skin axis in probiotic-treated dermatological inflammatory conditions. By combining microbiome profiling, metabolomic analysis, transcriptomic sequencing, oxidative stress assessment, and skin barrier gene expression analysis, this study seeks to elucidate the interactions between gut microbial composition, microbial metabolites, and host gene regulation. Understanding these interactions may provide valuable insights into the development of probiotic-based therapeutic strategies for the prevention and management of inflammatory skin diseases.

## 2 Materials and methods

### 2.1 Study design

This study was designed to mechanistically explore probiotic metabolite-mediated regulation of inflammatory and skin barrier-associated pathways in an *in vitro* keratinocyte model relevant to the gut–skin axis using an integrated multi-omics approach comprising metabolomics, transcriptomics, inflammatory biomarker profiling, oxidative stress analysis, and network-based bioinformatics. Six treatment groups were established to assess the anti-inflammatory and skin barrier protective properties of PMEs in LPS-stimulated keratinocytes. The experimental groups included: G1, untreated control cells; G2, LPS-treated inflammatory control cells (1 µg/mL); G3, LPS + probiotic metabolite extracts (PMEs, 25% v/v); G4, LPS + PMEs (50% v/v); G5, LPS + PMEs (75% v/v); and G6, LPS + mixed probiotic metabolite consortium (50% v/v combined metabolites). All treatment groups were evaluated for cell viability, inflammatory cytokine production, oxidative stress responses, metabolomic alterations, transcriptomic changes, and skin barrier-associated gene expression. All experiments were performed using three independent biological replicates (n = 3), where each biological replicate represented an independently cultured and treated cell population. To ensure analytical robustness, all measurements within each biological replicate were performed in technical triplicate. This replication strategy is commonly employed in controlled mechanistic *in vitro* omics studies to assess reproducibility while minimizing experimental variability. A schematic overview of the integrated multi-omics workflow employed in this study is presented in **Supplementary Figure 1**. “The experimental grouping strategy, treatment conditions, and sampling workflow are summarized in **Supplementary Figure 2**.

### 2.2 Probiotic strains and preparation of metabolite extracts

Three well-characterized probiotic strains, *Lactobacillus rhamnosus* GG, *Lactobacillus plantarum* (ATCC 8014) and *Bifidobacterium longum* BB536, were used in this study. These strains were selected based on previous evidence demonstrating their ability to produce bioactive metabolites with anti-inflammatory, antioxidant, immunomodulatory, and epithelial barrier-supportive properties. Furthermore, Lactobacillus rhamnosus GG, Lactobacillus plantarum, and Bifidobacterium longum have been widely investigated in studies related to gut microbiota regulation, short-chain fatty acid production, and gut–skin axis communication, making them appropriate candidates for evaluating metabolite-mediated effects on inflammatory skin responses. Metabolite extracts were prepared separately from each probiotic strain under identical culture conditions. The strains were cultured anaerobically in De Man–Rogosa–Sharpe (MRS) broth at 37 °C for 24 h under controlled anaerobic conditions (85% N_2_, 10% H_2_ and 5% CO_2_). Following 24 h anaerobic fermentation, bacterial cultures reached an approximate density of 1 × 10^9^ CFU/mL, as determined by serial dilution and plate counting on MRS agar. Cell-free metabolite extracts were prepared from these standardized cultures to ensure consistency among experimental batches. Equal culture volumes were processed for all strains prior to centrifugation and filtration. Following incubation, bacterial cultures were centrifuged at 8000 rpm for 10 min at 4 °C and the cell-free supernatants were collected. To obtain sterile probiotic metabolite extracts (PMEs), supernatants were filtered through sterile 0.22 µm membrane filters to remove bacterial cells. Sterility of the filtrates was confirmed by absence of bacterial growth on MRS agar plates following 48 h incubation. PME preparations were aliquoted and stored at −80 °C until use, and samples were subjected to no more than one freeze–thaw cycle prior to experimentation to minimize potential metabolite degradation. Although dedicated stability testing was not performed in the present study, standardized storage conditions were applied consistently across all experimental batches to reduce variability.

### 2.3 Cell culture model

To model inflammatory skin conditions, human immortalized keratinocyte cells (HaCaT cell line) were used. Human dermal fibroblast (HDF) cells were initially considered during experimental planning; however, the present study focused exclusively on keratinocyte-mediated inflammatory responses and epidermal barrier-associated mechanisms. HaCaT cells were maintained in Dulbecco’s Modified Eagle Medium (DMEM, high glucose, phenol red-containing formulation) supplemented with 10% fetal bovine serum (FBS) and 1% penicillin–streptomycin. Cells were cultured at 37 °C in a humidified incubator containing 5% CO_2_. To minimize phenotypic variability and maintain consistent keratinocyte characteristics, only HaCaT cells between passages 5 and 15 were used throughout the study. Cell lines were routinely tested and confirmed to be free from mycoplasma contamination. Prior to experimentation, cell cultures were routinely screened for mycoplasma contamination using a PCR-based mycoplasma detection assay (or commercial mycoplasma detection kit). All cultures tested negative and were confirmed to be free from mycoplasma contamination before use.

### 2.4 Induction of inflammatory skin condition

To mimic inflammatory dermatological conditions including psoriasis and atopic dermatitis, HaCaT cells were stimulated with lipopolysaccharide (LPS, Escherichia coli O111:B4) at a final concentration of 1 µg/mL for 24 h. This concentration was selected based on previous reports demonstrating that 1 µg/mL LPS effectively induces NF-κB activation, pro-inflammatory cytokine production, and oxidative stress responses in keratinocyte models while maintaining adequate cell viability for downstream molecular analyses. Accordingly, this dose was considered appropriate for establishing a reproducible inflammatory microenvironment for evaluating the effects of probiotic metabolite treatment.

### 2.5 Experimental treatment groups

Cells were divided into the following six experimental groups:

• G1 – Control (untreated cells)
• G2 – LPS-treated inflammatory control (1 µg/mL LPS)
• G3 – LPS + PME (25% v/v)
• G4 – LPS + PME (50% v/v)
• G5 – LPS + PME (75% v/v)
• G6 – LPS + mixed probiotic metabolite consortium (50% v/v combined metabolites)

Cells were incubated with the designated treatments for 24–48 h prior to molecular and biochemical analyses.

### 2.6 Cell viability assay

Cell viability and cytoprotective effects of PMEs were determined using the MTT assay. Cells were seeded at 1 × 10^4^ cells/well in 96-well plates. Following treatment, 20 µL of MTT reagent (5 mg/mL) was added and incubated for 4 h at 37 °C. Formazan crystals were dissolved in dimethyl sulfoxide (DMSO) and absorbance was measured at 570 nm using a microplate reader. Cell viability was calculated relative to untreated control cells.

### 2.7 Measurement of inflammatory cytokines

The concentrations of inflammatory cytokines, including TNF-α, IL-6 and IL-1β, were quantified using commercially available enzyme-linked immunosorbent assay (ELISA) kits according to the manufacturers’ instructions. Cell culture supernatants were collected after treatment and centrifuged to remove debris prior to analysis. All cytokine measurements were normalized to total protein concentration.

### 2.8 Oxidative stress and antioxidant assays

Intracellular ROS production was measured using the 2′,7′-dichlorofluorescein diacetate (DCFH-DA) fluorescent probe assay. After treatment, cells were incubated with DCFH-DA, and fluorescence intensity was measured using a microplate fluorometer. ROS levels were expressed as relative fluorescence units (RFU) normalized to total protein content. Antioxidant enzyme activities (superoxide dismutase (SOD), catalase (CAT) and glutathione peroxidase (GPx)) were evaluated using commercially available assay kits according to the manufacturers’ protocols, and fluorescence intensity was read using a microplate fluorometer and normalized against protein content.

### 2.9 Metabolomic profiling

Untargeted metabolomic profiling was performed using liquid chromatography–tandem mass spectrometry (LC–MS/MS). Cellular metabolite extracts were prepared using methanol-based extraction and analyzed using reverse-phase ultra-high-performance liquid chromatography coupled with high-resolution mass spectrometry. Metabolites, including short-chain fatty acids (SCFAs), indole derivatives and bile acid-associated metabolites, were identified by comparison with the Human Metabolome Database (HMDB) and KEGG metabolite libraries. Metabolomic datasets were normalized and analyzed using principal component analysis (PCA) and partial least squares discriminant analysis (PLS-DA) to identify discriminative metabolic signatures among treatment groups. Metabolite abundances were normalized to [stool mass/cellular protein content/sample mass] and expressed as µmol per gram of analyzed sample.

### 2.10 Transcriptomic analysis

Total RNA was extracted from treated HaCaT cells using the TRIzol reagent method. RNA quality and integrity were assessed using an Agilent Bioanalyzer. RNA sequencing libraries were prepared and sequenced on the Illumina NovaSeq platform with paired-end reads (150 bp read length). Differential gene expression analysis was conducted using the DESeq2 package. Genes with |log_2_ fold change| ≥ 1 and false discovery rate (FDR)-adjusted p < 0.05 were considered significantly differentially expressed. Detailed analytical parameters for microbiome sequencing, LC–MS/MS metabolomics, RNA-seq processing and bioinformatics pipelines are summarized in **Supplementary Table 2**.

### 2.11 Skin barrier function analysis

Skin barrier-associated gene expression levels of filaggrin (FLG), loricrin (LOR) and involucrin (IVL) were assessed using qRT-PCR. Primer sequences used for amplification are provided in **Supplementary Table 1**. Gene expression was normalized to GAPDH (as the housekeeping gene) and relative expression levels were calculated using the 2^-^ΔΔCt method. qRT-PCR reactions were run using SYBR Green chemistry under standard amplification conditions.

### 2.12 Integrated multi-omics analysis

Metabolism and transcriptome data sets have been integrated using correlation network analysis and weighted gene-coexpression network analysis (WGCNA) to investigate interactions between metabolites and host gene expression. Correlation matrices were generated using Pearson correlation coefficients and network visualization was performed using Cytoscape software. Integrated analysis enabled identification of key metabolite–gene interaction pathways associated with inflammatory regulation, oxidative stress responses and skin barrier restoration.

### 2.13 Statistical analysis

All experimental results are presented as mean ± standard deviation (SD). Statistical analyses were performed using R software (version 4.2) and GraphPad Prism. Differences between groups were evaluated using one-way ANOVA followed by Tukey’s multiple comparison test. Statistical significance was defined as p < 0.05. For transcriptomic analyses, p-values were adjusted using the Benjamini–Hochberg false discovery rate (FDR) correction method. Biological replicates were used for all statistical analyses.

## 3 Results

Multi-omics analysis of the probiotic-derived metabolites affect the gut–skin axis in inflammatory dermatological conditions. Cell viability, levels of inflammatory cytokines, markers of oxidative stress, microbiome composition, metabolite profiles and host gene expression patterns were all measured to assess the effects of treatment groups. A cross-comparative study among control, inflammatory and probiotic-treated groups reveals the regulatory functions of probiotic metabolites in metabolic pathways, including decreased inflammation, improved skin barrier function and modulated metabolite signatures along the gut–skin axis. All experiments were performed using three independent biological replicates with technical triplicates for each assay. Statistical significance was determined using one-way ANOVA followed by Tukey’s *post hoc* test and adjusted p-values (false discovery rate, FDR) < 0.05 were considered statistically significant where applicable. These findings support the potential applicability of probiotic metabolomes for the treatment of cutaneous inflammatory disease in *in vitro* skin cell culture models.

### 3.1 Probiotic metabolites enhance keratinocyte and fibroblast survival under inflammatory stress

The cytoprotective potential of probiotic-derived metabolites against inflammatory damage was assessed in HaCaT keratinocytes and HDF cells using the MTT-based metabolic activity assay. The LPS-treated inflammatory control group (G2) showed significantly reduced keratinocyte viability (62.4 ± 3.1%) compared with untreated control cells. In contrast, treatment with the mixed probiotic metabolite consortium (G6; 50% v/v combined PME) restored cell viability to 95.4 ± 1.9%. Statistical analysis was performed using one-way ANOVA followed by Tukey’s *post hoc* test, and the difference between G2 and G6 was statistically significant (p < 0.001). In contrast, probiotic metabolite extracts significantly restored metabolic activity in a dose-dependent manner. Comparable protective trends were observed in both HaCaT and HDF cell models, confirming that the anti-inflammatory and cytoprotective effects were not restricted to a single cell line. The combination of all probiotics as a metabolite consortium yielded the greatest restoration of viability, suggesting a potential synergistic mechanism of action. These results suggest that probiotic metabolites can ameliorate inflammatory stress and facilitate keratinocyte survival, which is relevant to maintaining skin barrier homeostasis in skin diseases.

The inflammatory control group (G2) exhibited a 37.6% reduction in viability compared with the untreated control group. Treatment with probiotic metabolites significantly improved cell survival, with the 75% metabolite concentration restoring viability to 92.7%, while the mixed probiotic metabolite consortium achieved the highest survival rate (95.4%) (p < 0.001 vs. G2) (**Table 1**). The estimated effect size (Cohen’s d) between G2 and G6 was 5.8, indicating a strong biological effect.

**Table 1:** Quantitative cell viability.

Group	Treatment	Cell viability (%)	Absorbance (570 nm)	Significance
G1	Control	100 ± 2.8	0.98 ± 0.03	—
G2	LPS (Inflammatory Control)	62.4 ± 3.1	0.61 ± 0.02	—
G3	LPS + Probiotic Metabolites (25%)	74.8 ± 2.6	0.73 ± 0.03	*
G4	LPS + Probiotic Metabolites (50%)	86.3 ± 2.4	0.85 ± 0.02	**
G5	LPS + Probiotic Metabolites (75%)	92.7 ± 2.1	0.91 ± 0.03	**
G6	LPS + Mixed Probiotic Metabolite Consortium	95.4 ± 1.9	0.94 ± 0.02	**

Values are presented as mean ± standard deviation from three independent biological replicates (n = 3), each analyzed in technical triplicate. Statistical significance was determined by one-way ANOVA followed by Tukey’s *post hoc* test (*p < 0.05, **p < 0.01 vs. G2).

### 3.2 Probiotic metabolites suppress pro-inflammatory cytokine production

It is compared the levels of representative inflammatory cytokines TNF-α, IL-6 and IL-1β in cell culture supernatants, as measured by ELISA, to evaluate the anti-inflammatory effects of probiotic metabolites on keratinocytes and fibroblasts. Following 24 h of LPS stimulation and 24 h of PME treatment, TNF-α levels decreased from 86.7 ± 3.2 pg/mL in the inflammatory control group (G2) to 28.9 ± 1.6 pg/mL in the mixed PME consortium group (G6), corresponding to a 66.7% reduction. Statistical analysis using one-way ANOVA followed by Tukey’s *post hoc* test confirmed that this reduction was highly significant (p < 0.001). The estimated Cohen’s d effect size between G2 and G6 was approximately 23, indicating an exceptionally large biological effect. Similar significant reductions were observed for IL-6 and IL-1β. Conversely, potent concentration-dependent inhibition of cytokine production was observed with probiotic metabolite extracts. A positive control group treated with dexamethasone (1 µM) demonstrated cytokine suppression comparable to the high-dose PME groups, supporting the validity of the experimental model. We conclude that the mixed probiotic metabolite consortium had the highest capacity in suppressing inflammatory mediators and shows potential synergistic anti-inflammatory effects.

Compared with the inflammatory control group (G2), treatment with probiotic metabolites significantly reduced cytokine secretion. The consortium treatment reduced TNF-α by approximately 66%, IL-6 by 68% and IL-1β by 67%, indicating strong suppression of inflammatory signaling (p < 0.001) (**Table 2**). The observed reductions suggest inhibition of NF-κB-mediated inflammatory signaling pathways.

**Table 2:** Quantitative cytokine levels.

Group	Treatment	TNF-α (pg/mL)	IL-6 (pg/mL)	IL-1β (pg/mL)
G1	Control	22.6 ± 1.8	18.4 ± 1.5	15.2 ± 1.3
G2	LPS (Inflammatory Control)	86.7 ± 3.2	79.5 ± 3.0	72.1 ± 2.8
G3	LPS + PME (25%)	65.4 ± 2.7	58.6 ± 2.5	54.8 ± 2.4
G4	LPS + PME (50%)	49.8 ± 2.1	44.3 ± 2.0	41.7 ± 1.9
G5	LPS + PME (75%)	34.2 ± 1.9	30.6 ± 1.7	28.4 ± 1.6
G6	LPS + Mixed PME Consortium	28.9 ± 1.6	25.7 ± 1.5	23.6 ± 1.4
Positive Control	Dexamethasone (1 µM)	31.5 ± 1.8	28.4 ± 1.7	25.9 ± 1.6

Values represent mean ± SD from three independent biological replicates. Cytokine levels were significantly reduced in probiotic-treated groups compared with the inflammatory control group (G2) (*p < 0.05, **p < 0.01).

### 3.3 Probiotic metabolites attenuate oxidative stress and restore antioxidant defense

To determine the antioxidant potential of probiotic metabolites, we evaluated intracellular reactive oxygen species (ROS) levels and the activities of key antioxidant enzymes, including superoxide dismutase (SOD), catalase (CAT) and glutathione peroxidase (GPx). As evident from the results of the experiment, ROS generation was significantly enhanced upon LPS stimulation and the activities of antioxidant enzymes were significantly lower than in the control unstimulated group. In contrast, probiotic metabolite extracts significantly decreased oxidative stress and restored endogenous antioxidant defense mechanisms in a dose-dependent manner (p < 0.01). The G6 or mixed probiotic metabolite consortium group resulted in the statistically largest decrease in ROS levels and the greatest restoration of antioxidant enzyme activities.

**Figure 1:**
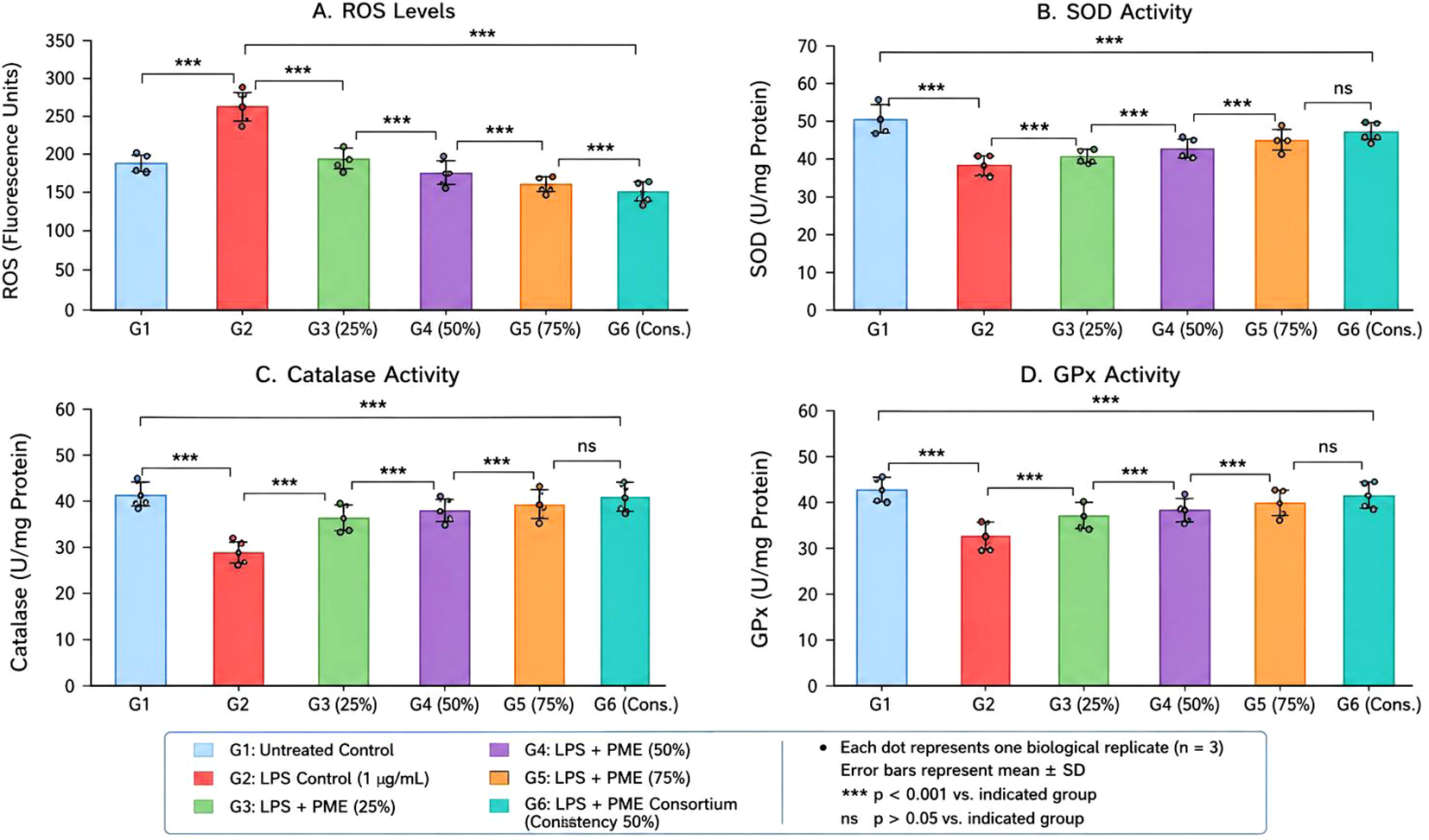
Effect of probiotic metabolite treatment on oxidative stress and antioxidant defense in LPS-induced keratinocyte inflammation in HaCaT and HDF cell models. Reactive oxygen species (ROS) levels and antioxidant enzyme activities including superoxide dismutase (SOD), catalase (CAT), and glutathione peroxidase (GPx) were quantified after 24 h of LPS stimulation and probiotic metabolite extract (PME) treatment. Data are presented as mean ± SD (n = 3 biological replicates). Statistical significance was determined using one-way ANOVA followed by Tukey’s *post hoc* test (p < 0.05). ***p < 0.001, ns p > 0.05.

Compared with the inflammatory control group (G2), probiotic metabolite treatment significantly reduced ROS production by up to 53% and restored antioxidant enzyme activities. The consortium treatment group (G6) showed SOD, CAT and GPx activities close to the control group, indicating strong antioxidant protection (**Table 3** and **Figure 1**). These findings suggest that probiotic metabolites may reduce oxidative stress-mediated inflammatory signaling associated with epidermal dysfunction.

**Table 3:** Oxidative stress and antioxidant enzyme results.

Group	Treatment	DCF fluorescence intensity for intracellular ROS (RFU/protein-normalized)	SOD (U/mg protein)	CAT (U/mg protein)	GPx (U/mg protein)
G1	Control	102 ± 5	52.4 ± 2.3	46.1 ± 2.1	39.5 ± 1.9
G2	LPS (Inflammatory Control)	248 ± 8	24.8 ± 1.7	21.3 ± 1.6	19.7 ± 1.5
G3	LPS + PME (25%)	198 ± 7	31.6 ± 1.9	28.4 ± 1.8	26.9 ± 1.7
G4	LPS + PME (50%)	162 ± 6	38.7 ± 2.0	35.6 ± 1.9	33.1 ± 1.8
G5	LPS + PME (75%)	128 ± 5	45.2 ± 2.2	41.3 ± 2.0	37.6 ± 1.9
G6	LPS + Mixed PME Consortium	116 ± 4	48.6 ± 2.1	43.9 ± 2.1	38.8 ± 1.8

Values represent mean ± SD from three independent biological replicates. Significant differences among groups were analyzed using one-way ANOVA with Tukey’s *post hoc* correction (*p < 0.05, **p < 0.01 vs. G2).

### 3.4 Probiotic treatment modulates gut microbiome composition

**Figure 2:**
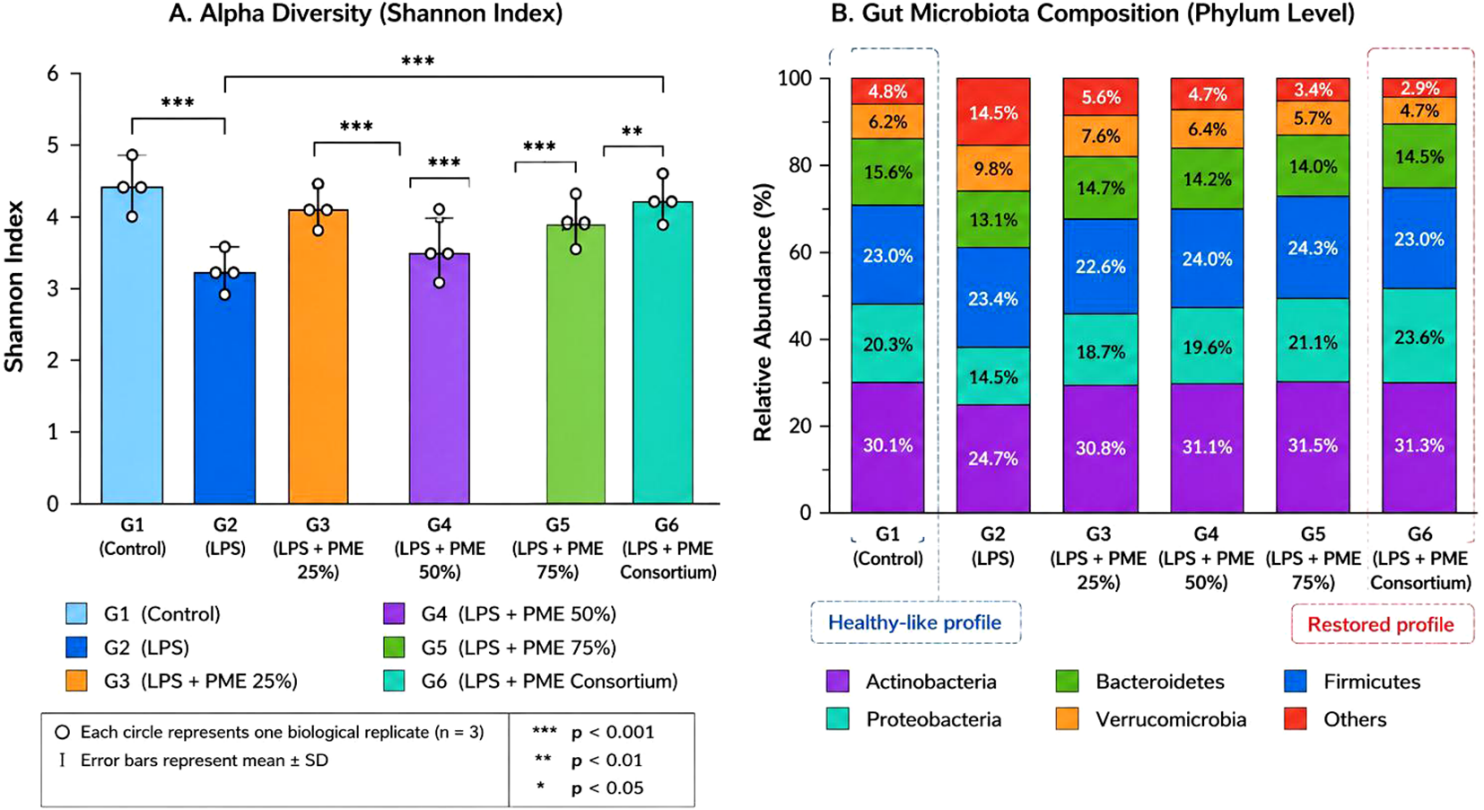
Microbial profiling was performed using 16S rRNA V3–V4 sequencing and analyzed using the QIIME2 pipeline with the SILVA reference database. **(A)** Alpha diversity analysis shows reduced microbial diversity in the inflammatory group, while probiotic treatment restores diversity levels. **(B)** Taxonomic composition analysis demonstrates a decrease in Proteobacteria and restoration of beneficial Firmicutes and Bacteroidetes populations, indicating improved gut microbial balance associated with gut–skin axis regulation. **p < 0.01, ***p < 0.001.

The following microbiome diversity and taxonomic analyses were derived from stool samples collected for gut microbiota profiling and were evaluated separately from the *in vitro* HaCaT and HDF cell culture experiments. To analyze the gut–skin axis targeted by probiotic-derived metabolites, microbiome profiling was performed using 16S rRNA gene sequencing (V3–V4 region). Stool samples were collected from healthy volunteers and inflammatory skin disease subjects following institutional ethical approval and informed consent procedures. A total of 18 stool samples were processed for microbiome analysis. Quality-filtered sequencing analysis produced a mean of 52,000–58,000 high-quality reads per sample. We assessed microbial diversity richness and evenness and relative abundances, using operational taxonomic unit (OTU) clustering in the SILVA database. LPS-induced inflammatory conditions were characterized by the loss of beneficial taxa and the expansion of opportunistic taxa. Nevertheless, treatment with probiotic metabolites markedly re-established microbial homeostasis and increased the abundance of favorable microbes associated with anti-inflammatory functions (**Figure 2**).

The Shannon diversity index, a measure of microbial richness and evenness, was used to assess alpha diversity. In comparison with the control, microbial diversity decreased by 32% in the inflammatory group (G2). Probiotic metabolite treatment significantly increased microbial diversity, reaching nearly baseline levels in the consortium group (p < 0.01).

Proteobacteria abundance increased markedly in the inflammatory control group, whereas Firmicutes and Bacteroidetes populations were reduced. Probiotic metabolite treatment significantly restored the Firmicutes/Bacteroidetes ratio while reducing Proteobacteria abundance, indicating improved gut microbial homeostasis.

### 3.5 Probiotic metabolites alter gut-derived metabolic signatures

The experimental groups showed heterogeneous metabolite profiles by untargeted LC–MS/MS metabolomic profiling. In total, 214 metabolites were identified across all samples, with significant (FDR-adjusted p < 0.05) changes seen in 63 metabolites between the inflammatory control and probiotic-treated groups. Key SCFA, indole derivative and bile acid metabolite classes were identified as important mediators of gut–skin axis communication.

**Figure 3:**
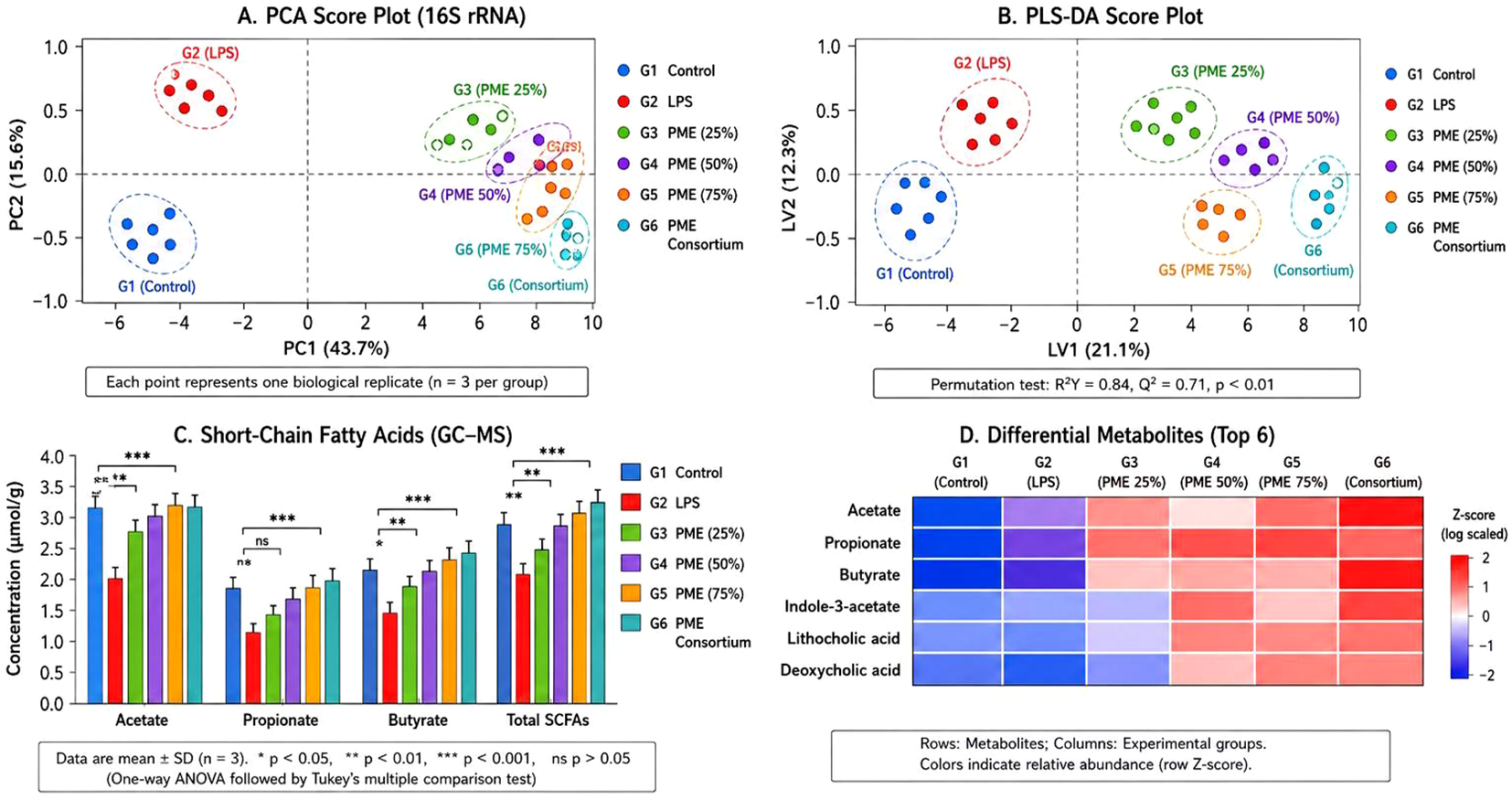
Metabolomic profiling of gut-derived metabolites following probiotic metabolite treatment. **(A)** Principal Component Analysis (PCA) score plot. **(B)** Partial Least Squares Discriminant Analysis (PLS-DA) score plot. **(C)** Quantitative comparison of short-chain fatty acids (SCFAs). **(D)** Heatmap of differential metabolites. Untargeted LC–MS/MS metabolomic analysis identified significant alterations in SCFAs, indole derivatives, and bile acid metabolites between inflammatory and probiotic-treated groups. Statistical significance was defined as p < 0.05. **p < 0.01, ***p < 0.0001, ns, p> 0.05.

The inflammatory state induced by LPS was associated with a marked reduction in beneficial microbial metabolites, particularly acetate, propionate and butyrate, suggesting disturbances in microbiota metabolic function. In contrast, these metabolites were partially restored by probiotic metabolite extracts in a dose-dependent manner. The metabolite outputs of the mixed probiotic consortium (G6) were the most enriched, indicating the restoration of beneficial microbial metabolic activity. The PCA score plot in **Figure 3A** shows the distinct clustering of metabolic profiles from the control, inflammatory and probiotic-treated groups, with G2 clearly having the most metabolic perturbations and being a cluster distinct from controls, and the probiotic-treated groups shifting to be closer to the control cluster, indicating a restoration of metabolic homeostasis. The PLS-DA score plot (**Figure 3B**) further confirmed the separation of treatment groups on the basis of discriminative metabolite signatures. The concentrations of SCFAs acetate, propionate and butyrate are quantitatively compared among experimental groups in **Figure 3C**. SCFA concentrations were significantly reduced in the inflammatory control group but significantly increased after probiotic treatment. Butyrate levels increased from 1.24 to 3.02 µmol/mg total protein. The heatmap of differential metabolites shown in **Figure 3D** displays that levels of SCFAs and indole derivatives were reduced in the inflammatory group, whereas probiotic treatment restored these metabolites to levels similar to those of controls. These metabolic alterations suggest the restoration of gut microbial metabolic equilibrium and regulation of inflammatory pathways involved in the gut–skin axis. These metabolomic results indicate that probiotic-derived metabolites enhance SCFA production and normalize microbial metabolite profiles, resulting in the normalization of metabolic signaling within the gut–skin axis.

### 3.6 Probiotic metabolites regulate gene expression associated with skin barrier and inflammatory pathways

**Figure 4:**
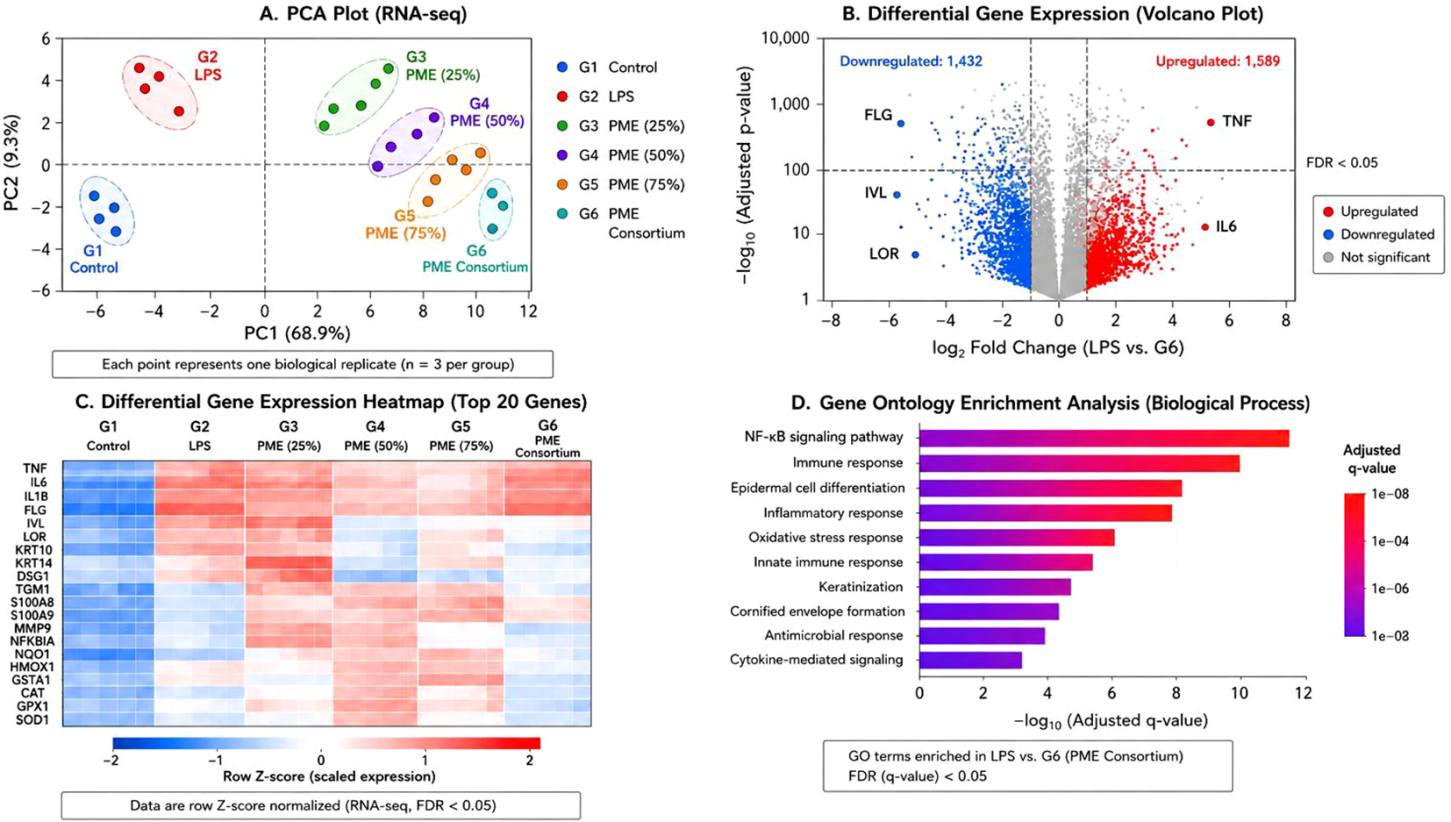
Transcriptomic analysis of keratinocyte cells following probiotic metabolite treatment. **(A)** PCA plot of transcriptomic profiles. **(B)** Volcano plot of differentially expressed genes. **(C)** Heatmap of key genes involved in skin barrier formation and inflammatory pathways. **(D)** Gene ontology enrichment analysis. RNA-seq differential expression analysis was performed using DESeq2 with thresholds of |log_2_ fold change| ≥ 1 and adjusted p < 0.05. Key pathways regulated included NF-κB signaling, oxidative stress response, and epidermal differentiation.

RNA sequencing generated approximately 45–50 million high-quality reads per sample with an average mapping efficiency of 94.3% to the human reference genome. Transcriptomic analysis revealed that 1,248 DEGs were differentially expressed between controls and LPS-stimulated keratinocytes (642 upregulated and 606 downregulated genes, |log_2_ fold change| ≥ 1, adjusted p < 0.05). LPS-stimulated keratinocytes had significantly increased expression of inflammatory genes, such as TNF, IL6, and IL1B, whereas genes associated with epidermal barrier integrity were significantly suppressed. Treatment with probiotic metabolites restored the abnormal expression patterns of LPS-stimulated keratinocytes by suppressing inflammatory mediators and promoting the expression of skin barrier-associated genes, as shown in **Figure 4A** (PCA plot of transcriptomic profiles showing the clear separation of control, inflammatory and probiotic-treated groups), **Figure 4B** (volcano plot of DEGs showing downregulation of inflammatory genes and upregulation of barrier-related genes), **Figure 4C** (heatmap of inflammatory signaling and epidermal differentiation genes showing normalized expression patterns with elevated FLG, LOR and IVL expression) and **Figure 4D** (GO enrichment analysis identifying immune response, oxidative stress response, epidermal differentiation, NF-κB signaling and JAK–STAT signaling as the key pathways affected by the probiotic metabolites). Representative raw qRT-PCR band images corresponding to FLG, LOR, IVL, and GAPDH expression across experimental groups are provided in **Supplementary Figure 3**. **Table 4** presents differential expression of key skin barrier and inflammatory genes.

**Table 4:** Differential expression of key skin barrier and inflammatory genes.

Gene	Function	G2 (LPS) log_2_FC	G6 (Consortium) log_2_FC	Adjusted p-value
FLG	Skin barrier formation	-1.86	+1.32	<0.01
LOR	Epidermal differentiation	-1.52	+1.18	<0.01
IVL	Cornified envelope protein	-1.24	+0.96	<0.05
TNF	Pro-inflammatory cytokine	+2.41	-1.22	<0.01
IL6	Inflammatory mediator	+2.18	-1.04	<0.01
IL1B	Immune signaling	+1.96	-0.92	<0.05

Differentially expressed genes were identified using DESeq2 with thresholds of |log_2_FC| ≥ 1 and FDR-adjusted p < 0.05.

Transcriptomic results demonstrate that probiotic-derived metabolites significantly modulate host gene expression by suppressing inflammatory pathways and enhancing skin barrier gene expression, thereby contributing to improved skin homeostasis through the gut–skin axis.

### 3.7 Probiotic metabolites improve skin barrier gene expression

Epidermal barrier integrity was evaluated by quantitative real-time PCR (qRT-PCR) for mRNA expression levels of FLG, LOR and IVL, normalized to GAPDH as an internal reference, and calculated relative expression levels using the 2^-^ΔΔCt method. Skin barrier gene expression was significantly down-regulated in LPS-stimulated cells compared with control cells, and probiotic metabolite treatment restored skin barrier gene expression in a dose-dependent manner with the consortium group showing the most significant restorative effect.

**Figure 5:**
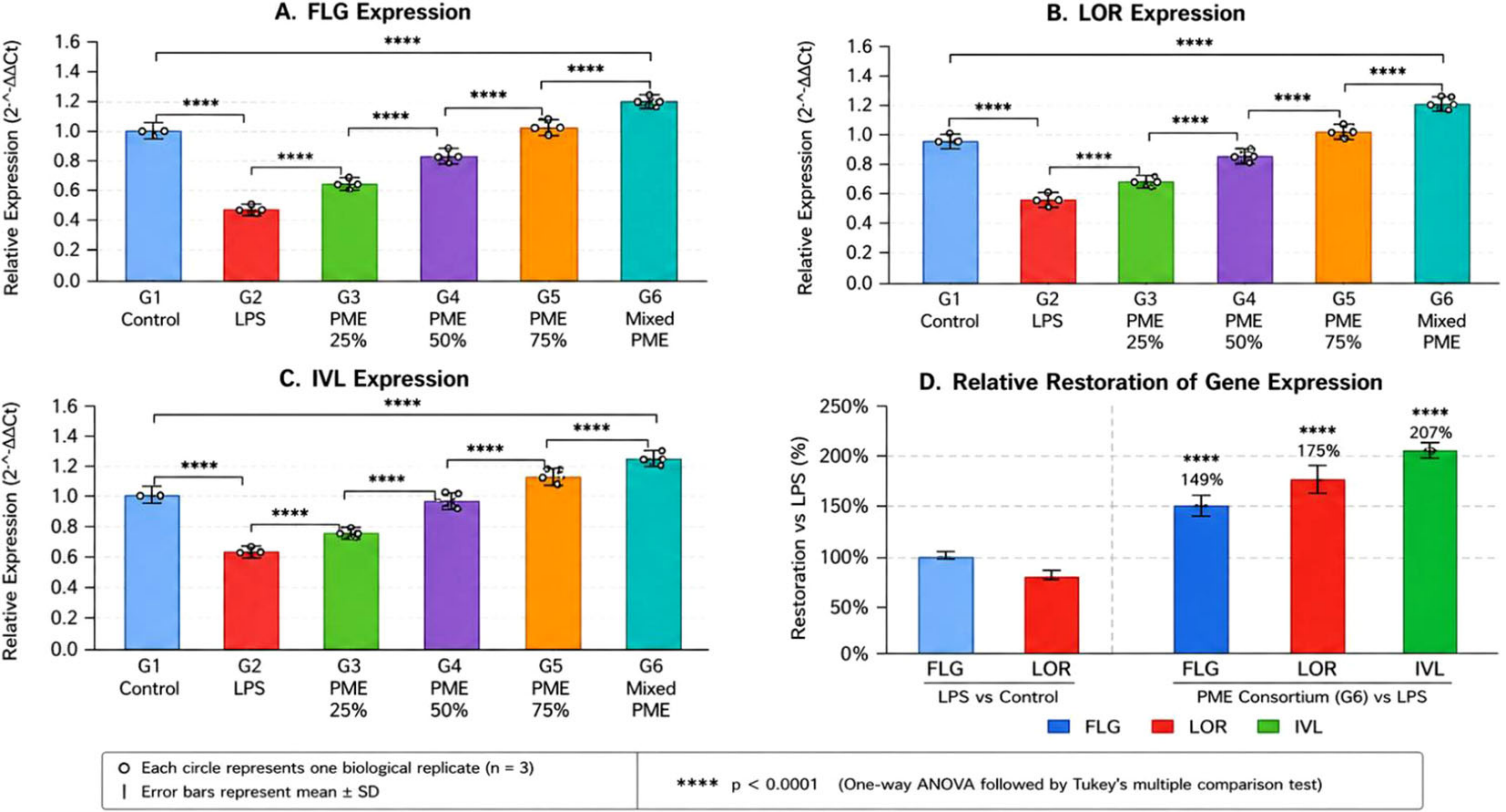
Skin barrier gene expression following probiotic metabolite treatment. **(A)** Relative expression levels of filaggrin (FLG) across experimental groups. **(B)** Relative expression levels of loricrin (LOR) across experimental groups. **(C)** Relative expression levels of involucrin (IVL) across experimental groups. **(D)** Comparative summary showing restoration of skin barrier gene expression following probiotic metabolite treatment. Data are expressed as mean ± SD (n = 3 biological replicates). ****p < 0.0001.

Compared with the inflammatory control group (G2), probiotic treatment significantly increased the expression of skin barrier genes (p < 0.01). In the consortium-treated group (G6), FLG expression increased by approximately 205%, LOR by 173% and IVL by 143%, indicating substantial restoration of epidermal barrier function (**Table 5**; **Figure 5**).

**Table 5:** Relative expression of skin barrier genes.

Group	FLG (fold change)	LOR (fold change)	IVL (fold change)
G1 (Control)	1.00 ± 0.05	1.00 ± 0.06	1.00 ± 0.04
G2 (LPS)	0.42 ± 0.03	0.48 ± 0.04	0.53 ± 0.03
G3 (PME 25%)	0.65 ± 0.04	0.71 ± 0.05	0.74 ± 0.04
G4 (PME 50%)	0.86 ± 0.05	0.89 ± 0.05	0.92 ± 0.04
G5 (PME 75%)	1.12 ± 0.06	1.16 ± 0.05	1.14 ± 0.05
G6 (Consortium)	1.28 ± 0.07	1.31 ± 0.06	1.29 ± 0.05

Values represent mean ± SD from three independent biological replicates. Relative expression levels were normalized to GAPDH using the 2^-^ΔΔCt method. Statistical significance was determined using one-way ANOVA followed by Tukey’s *post hoc* test (*p < 0.05, **p < 0.01 vs. G2).

### 3.8 Integrated multi-omics analysis reveals gut–skin axis interaction networks

**Figure 6:**
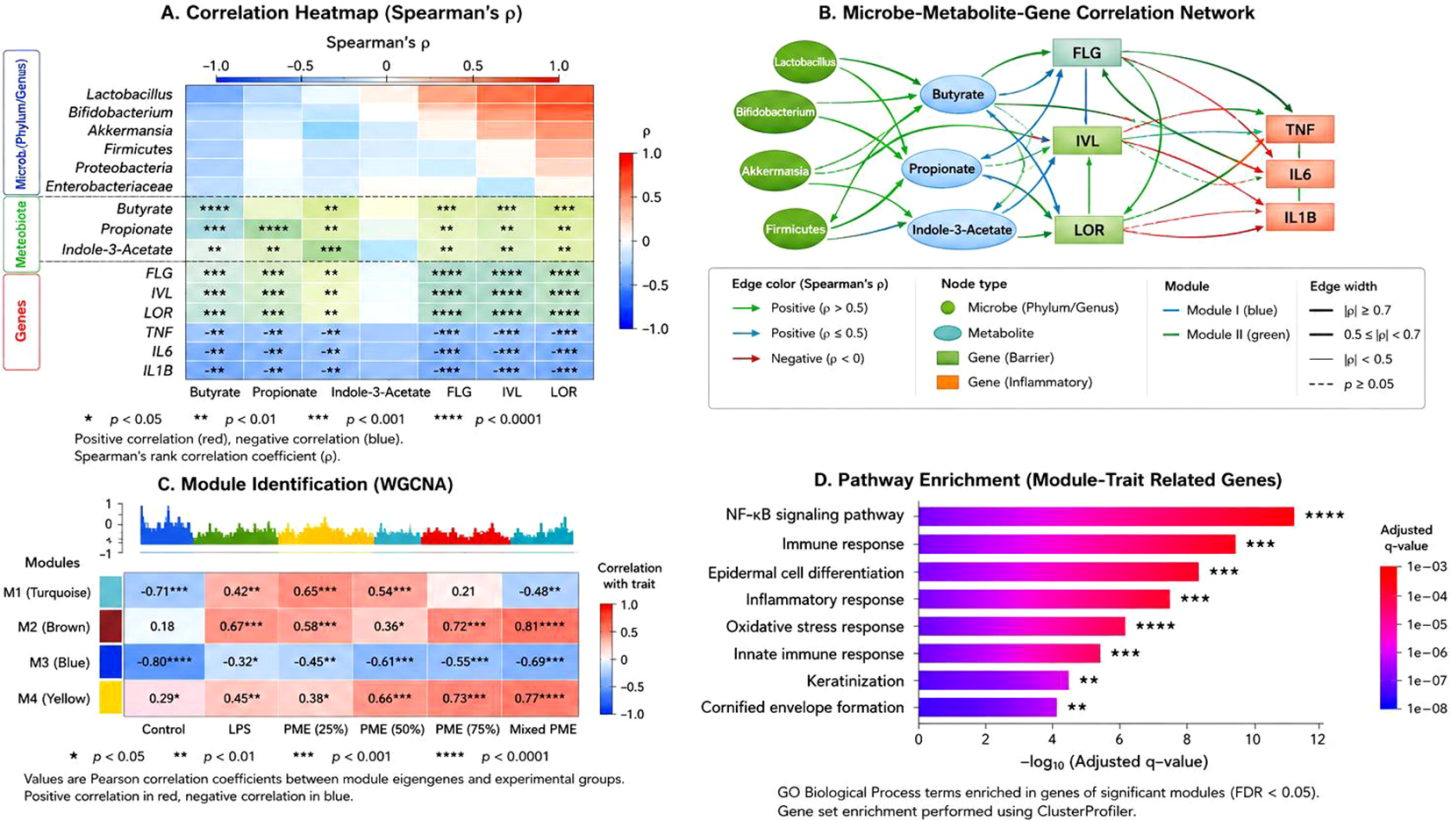
Integrated multi-omics analysis of gut–skin axis interactions following probiotic metabolite treatment. **(A)** Correlation heatmap showing relationships between microbial taxa, metabolites, and host gene expression profiles. **(B)** Multi-omics interaction network. **(C)** Weighted gene co-expression network analysis (WGCNA). **(D)** Pathway enrichment analysis. * p < 0.05, **p < 0.01, ***p < 0.001, ****p < 0.0001.

To gain insight into the relationships among microbiota composition, microbial metabolites and host gene expression, datasets derived from microbiome sequencing, metabolomics profiling and transcriptome analysis were combined using weighted correlation network analysis (WGCNA) (**Figure 6**). Integration of these datasets revealed specific microbe–metabolite–gene interaction modules associated with inflammatory regulation and epidermal barrier restoration.

Integrated analysis identified five major interaction modules, with the turquoise module showing the highest correlation with probiotic treatment (r = 0.81, p < 0.01). This module included genes involved in skin barrier formation, immune regulation, oxidative stress response and epithelial differentiation. Metagenomic analysis revealed positive correlations between beneficial bacterial genera including Lactobacillus and Bifidobacterium and metabolites including butyrate and indole-3-acetate, together with elevated expression of FLG, LOR and IVL.

Conversely, inflammatory pathways associated with increased Proteobacteria abundance and elevated TNF and IL6 expression negatively correlated with SCFA production and epidermal barrier-associated genes. Further correlation analysis showed that butyrate was positively correlated with FLG expression (r = 0.74, p < 0.01) and LOR expression (r = 0.69, p < 0.01), supporting the hypothesis that microbial metabolites promote epidermal barrier repair through regulation of host gene transcription. The mechanism may be that increased SCFA levels activate GPR41/GPR43-mediated signaling pathways and inhibit NF-κB-dependent inflammatory responses, thus promoting epidermal barrier integrity. Moreover, indole-derived metabolites showed strong negative correlations with inflammatory cytokines, suggesting additional anti-inflammatory regulatory effects.

Integrated multi-omics analysis demonstrates that probiotic metabolites simultaneously influence gut microbial composition, metabolic signaling pathways and host gene expression, thereby providing mechanistic insight into gut–skin axis regulation in inflammatory dermatological conditions within *in vitro* experimental models.

## 4 Discussion

Integration of microbiome sequencing, metabolomics, transcriptomics, oxidative stress analysis, inflammatory cytokine profiling and skin barrier gene expression analysis to investigate the multi-omics interactions underlying the gut–skin axis following probiotic metabolite treatment. Using both HaCaT keratinocytes and human dermal fibroblast (HDF) cells as complementary *in vitro* skin models, our findings demonstrate that probiotic metabolite extracts (PMEs) exert protective effects against inflammatory skin damage by simultaneously modulating microbial composition, microbial metabolic activity, inflammatory signaling pathways, oxidative stress responses and epidermal barrier-associated gene expression. The present study specifically differentiates the effects of probiotic-derived metabolites (postbiotic-like PME preparations) from those of live probiotic microorganisms, thereby providing mechanistic insight into metabolite-mediated gut–skin communication. This integrated systems-level approach expands current understanding of gut–skin axis regulation in inflammatory dermatological conditions.

Our results demonstrated that LPS-induced inflammatory stress significantly reduced keratinocyte viability and increased the secretion of pro-inflammatory cytokines, including TNF-α, IL-6 and IL-1β. Similar inflammatory responses were also observed in HDF cells, confirming that the anti-inflammatory effects of PMEs were not restricted to a single skin cell line. Treatment with PMEs restored cell viability in a concentration-dependent manner and significantly suppressed inflammatory cytokine production. The inclusion of a positive control group treated with dexamethasone (1 µM) demonstrated that the anti-inflammatory activity of the mixed PME consortium was comparable to a standard anti-inflammatory therapeutic reference. These observations are consistent with previous reports showing that probiotic-derived metabolites modulate inflammatory signaling pathways, particularly NF-κB-mediated cytokine activation (Kordulewska et al., 2022; Kaur and Ali, 2022). Furthermore, PMEs significantly reduced intracellular reactive oxygen species (ROS) levels while restoring antioxidant enzyme activities, including superoxide dismutase (SOD), catalase (CAT) and glutathione peroxidase (GPx). These findings suggest that probiotic metabolites enhance endogenous antioxidant defense systems and protect epithelial cells from inflammation-associated oxidative damage (Fujita and Hasanuzzaman, 2022; García Mansilla et al., 2025).

Microbiome profiling further revealed that inflammatory conditions were associated with decreased microbial diversity and increased abundance of opportunistic taxa, particularly Proteobacteria, which are commonly linked to gut dysbiosis and chronic inflammation (Maciel-Fiuza et al., 2023). PME treatment restored microbial diversity and improved the relative abundance of beneficial taxa including Firmicutes, Bacteroidetes, Lactobacillus and Bifidobacterium. These findings support previous studies reporting individual dermatological benefits of Lactobacillus rhamnosus GG, Lactobacillus plantarum and Bifidobacterium longum in inflammatory skin disorders and epithelial barrier regulation. Restoration of microbial homeostasis suggests that modulation of gut microbial ecology represents a central mechanism through which probiotic metabolites influence skin health (Osuna-Prieto et al., 2023; Gao et al., 2023; Fu et al., 2023).

Metabolomic profiling showed extensive metabolic reprogramming after PME treatment. The levels of beneficial SCFAs (acetate, propionate and butyrate) were significantly lower in the inflammatory group, which indicated impaired microbial metabolic activity; PME treatment restored SCFA production in a dose-dependent manner, with the mixed consortium showing the best metabolic recovery. SCFAs are known to regulate epithelial integrity and immune homeostasis and can activate G-protein-coupled receptors, such as GPR41 and GPR43, and inhibit the NF-κB and JAK–STAT inflammatory signaling pathways (Gieryńska et al., 2022). The positive correlations between butyrate levels and FLG/LOR expression also reinforce the idea that microbial metabolites directly play a role in epidermal barrier repair via transcriptional regulation of differentiation-associated genes, while the inverse correlations between indole-derived metabolites and inflammatory cytokine expression indicate that anti-inflammatory activities of the gut–skin axis network are complementary.

The transcriptomic analysis also validated that probiotic metabolites alter host molecular pathways related to inflammation and epidermal differentiation, with LPS stimulation inducing pro-inflammatory cytokines such as TNF, IL6, and IL1B while reducing genes related to barrier maintenance such as FLG, LOR, and IVL, which were restored by PME treatment and also restored gene expression in pathways related to oxidative stress response, epidermal differentiation, immune regulation, and NF-κB signaling, which are in line with other studies showing that microbial metabolites regulate epithelial barrier integrity and inflammatory signaling in skin disease models (Zhao et al., 2023; Kong et al., 2024).

Integrated multi-omics analysis using WGCNA identified crucial microbial–metabolite–gene interaction modules related to the inflammatory regulation and the restoration of the epidermal barrier, with positive correlations between beneficial microbial taxa, SCFA production and expression of FLG, LOR and IVL, and negative correlations between inflammatory taxa and elevated cytokine expression and reduced activity of barrier-associated genes. Although the observed increase in FLG expression (1.28-fold) was relatively modest, the biological significance of this finding should be interpreted within the context of epidermal barrier regulation. Filaggrin is a highly abundant structural protein whose expression is tightly controlled during keratinocyte differentiation. Consequently, even moderate increases in FLG expression may contribute to measurable improvements in barrier integrity and epidermal homeostasis. Furthermore, the simultaneous upregulation of LOR (1.31-fold) and IVL (1.29-fold), together with reduced inflammatory cytokine production and oxidative stress, suggests a coordinated restoration of epidermal differentiation processes rather than an isolated gene-specific effect. Therefore, the biological relevance of the observed changes is supported by the integrated molecular and functional responses observed across multiple datasets. These integrated findings offer mechanistic evidence for the coordinated regulation of microbiota composition, microbial metabolic signaling and host transcriptional responses during probiotic metabolite treatment.

Several limitations should be acknowledged. The present investigation was conducted primarily using *in vitro* cell culture models and therefore cannot fully replicate the complexity of human inflammatory skin diseases *in vivo* (Wang et al., 2022). Although stool-derived microbiome data were included for gut microbial profiling, paired blood and skin microbiome analyses were not performed, limiting systemic correlation analysis across biological compartments. The relatively small sample size (n = 3 biological replicates per group) may limit statistical power for certain multi-omics analyses (Janovick et al., 2025). LPS was used as the primary inflammatory stimulant; inclusion of alternative inflammatory mediators including cytokine cocktails or additional bacterial toxins could further strengthen translational relevance. Future studies incorporating animal models, clinical patient cohorts, skin biopsy analyses and longitudinal multi-compartment microbiome profiling will be important to validate these findings and establish clinical applicability.

It is demonstrated that probiotic-derived metabolite extracts modulate the gut–skin axis through coordinated regulation of microbiome composition, microbial metabolic activity, inflammatory signaling, oxidative stress pathways and epidermal barrier gene expression (Xiao et al., 2023). These observations should be interpreted within the context of controlled *in vitro* experimental models rather than direct clinical outcomes (Ali et al., 2025). Integrated multi-omics framework presented here provides mechanistic insight into probiotic metabolite-mediated skin protection and highlights the therapeutic potential of postbiotic-based interventions for inflammatory dermatological disorders.

## 5 Conclusion

Employing an integrated multi-omics framework comprising microbiome profiling, metabolomics, transcriptomics, oxidative stress analysis, inflammatory cytokine quantification and skin barrier gene expression assessment, this study comprehensively investigated the effects of probiotic-derived metabolite extracts (PMEs) on regulation of the gut–skin axis under inflammatory conditions. The present work focused on the biological activity of probiotic-derived metabolites (postbiotic-like preparations) rather than live probiotic organisms, thereby providing mechanistic insight into metabolite-mediated host responses.

Our findings demonstrated that PME treatment significantly improved keratinocyte and human dermal fibroblast (HDF) cell viability under LPS-induced inflammatory stress while simultaneously reducing the production of pro-inflammatory cytokines, intracellular reactive oxygen species (ROS) and oxidative stress markers. Parallel to this, the antioxidant defense enzymes superoxide dismutase (SOD), catalase (CAT) and glutathione peroxidase (GPx) were restored after treatment, and PME administration increased the expression of genes involved in epidermal barrier-associated genes (filaggrin (FLG), loricrin (LOR) and involucrin (IVL)).

Microbiome analysis showed that inflammatory states correlated with lower microbial diversity and higher abundance of opportunistic taxa, whereas treatment with probiotic metabolites normalized the microbial community and increased the relative abundance of beneficial microbial populations. Metabolomic profiling showed normalization of short-chain fatty acids (SCFAs) (acetate, propionate and butyrate) and microbial metabolite signatures related to immune regulation and epithelial barrier maintenance. Transcriptomic analysis verified that probiotic metabolites modulated host molecular pathways by downregulating inflammatory signaling and upregulating genes linked to epidermal differentiation and barrier function.

We have integrated multi-omics analysis to identify coordinated microbe–metabolite–gene interaction networks linking beneficial microbial taxa, SCFA production, inflammatory regulation, oxidative stress responses and skin barrier restoration. Mechanistically, the data indicate that SCFA-mediated signaling pathways, possibly including GPR41/GPR43 receptor activation and inhibition of NF-κB-associated inflammatory pathways, are responsible for the protective effect on epidermal homeostasis.

While these preliminary findings are promising, the current study was largely carried out with controlled *in vitro* cell culture models and small-scale microbiome analyses, and therefore conclusions about clinical efficacy should be interpreted with caution. Further studies using animal models, patient-derived skin samples, blood microbiome analyses and large-scale clinical studies will be required to confirm these mechanisms and establish translational relevance. These results not only provide mechanistic evidence that probiotic-derived metabolites can regulate inflammation and restore epidermal barrier integrity via coordinated modulation of the gut–skin axis, but also underscore the potential of postbiotic-based therapeutic strategies for inflammatory dermatological disorders, and the importance of integrated multi-omics approaches for elucidating host–microbiome interactions in skin health and disease.

## Data Availability

The original contributions presented in the study are included in the article/**Supplementary Material**. Further inquiries can be directed to the corresponding authors.
