# Supplementary figures and images for: Multi-omics integration of gut–skin axis in probiotic-treated dermatological conditions
Source: Front Cell Infect Microbiol. 2026 Jul 13;16:1834120. doi: 10.3389/fcimb.2026.1834120 (PMC13402189; doi:10.3389/fcimb.2026.1834120)

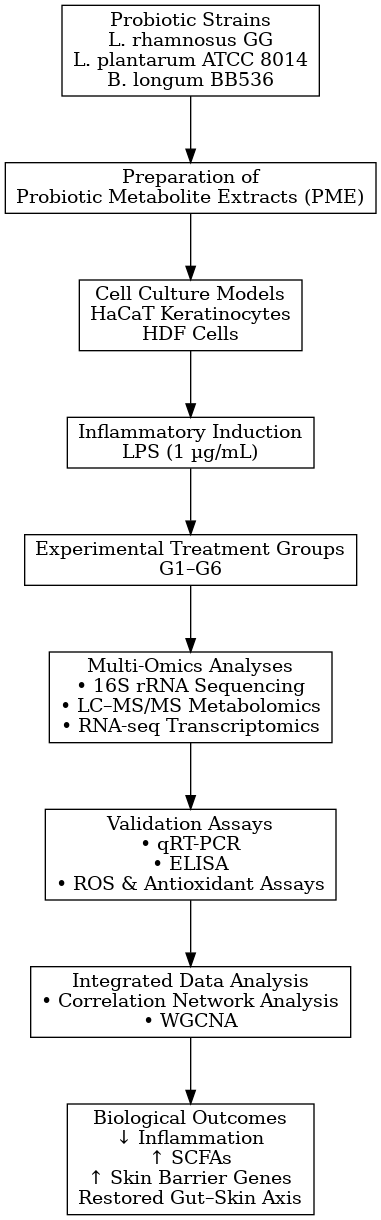

Supplement: Supplementary file 1 [file Image1.png]

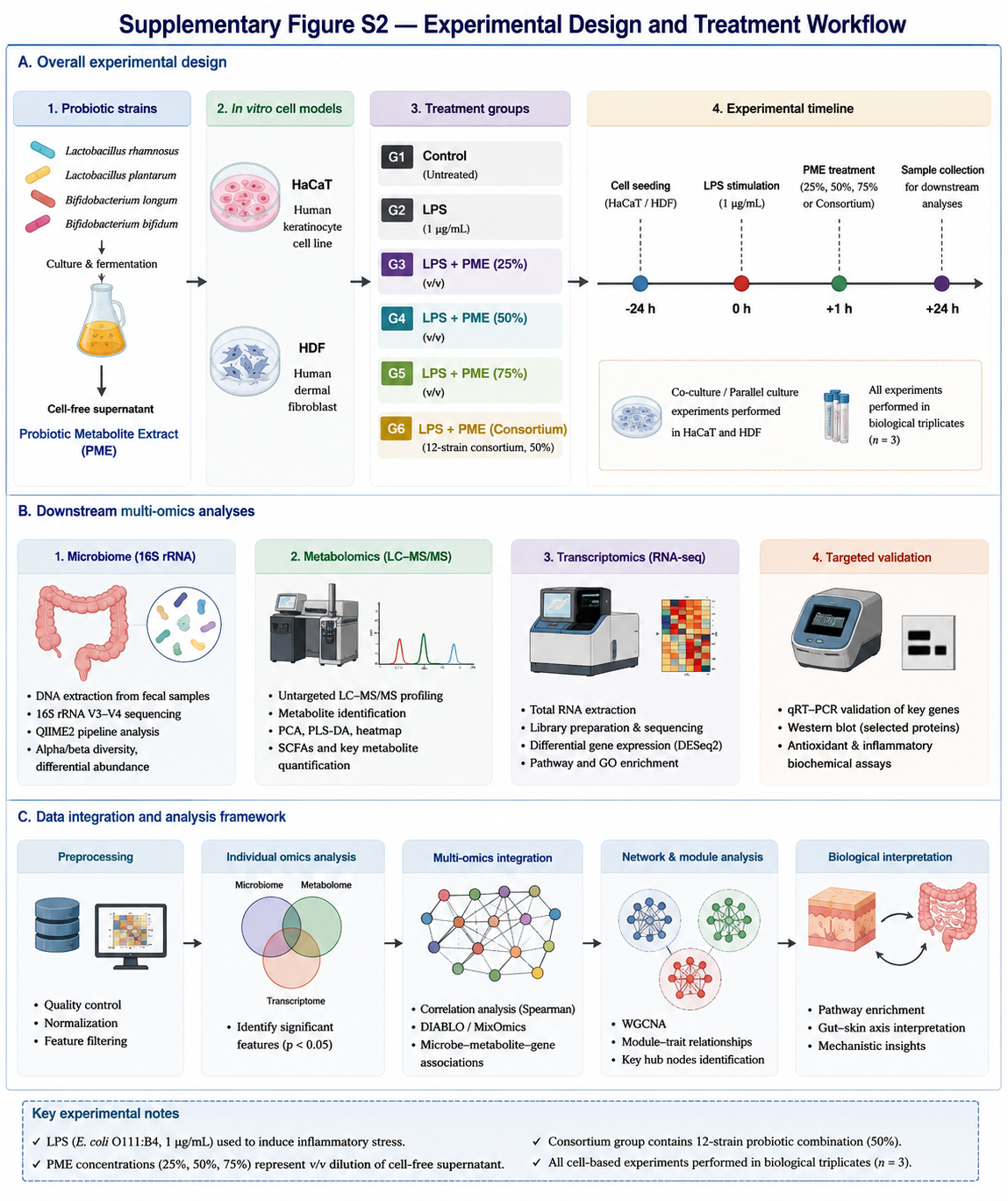

Supplement: Supplementary file 2 [file Image2.png]

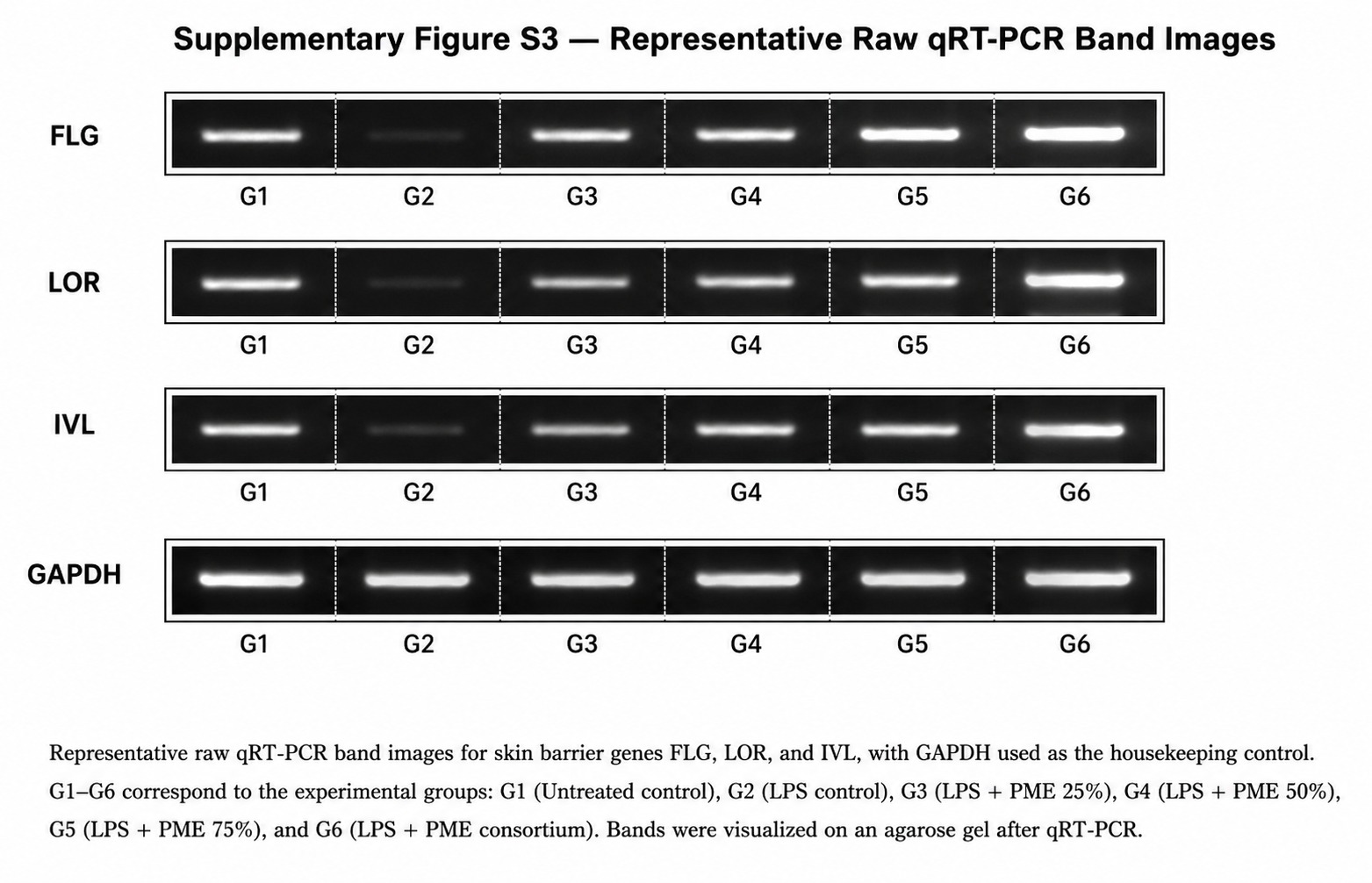

Supplement: Supplementary file 3 [file Image3.jpeg]
